# When Does Choice of Accuracy Measure Alter Imputation Accuracy Assessments?

**DOI:** 10.1371/journal.pone.0137601

**Published:** 2015-10-12

**Authors:** Shelina Ramnarine, Juan Zhang, Li-Shiun Chen, Robert Culverhouse, Weimin Duan, Dana B. Hancock, Sarah M. Hartz, Eric O. Johnson, Emily Olfson, Tae-Hwi Schwantes-An, Nancy L. Saccone

**Affiliations:** 1 Department of Genetics, Washington University, St. Louis, Missouri, United States of America; 2 Chinese Academy of Sciences, Key Laboratory of Brain Function and Disease, School of Life Sciences, University of Science and Technology of China, Hefei, Anhui, China; 3 Department of Psychiatry, Washington University, St. Louis, Missouri, United States of America; 4 Department of Medicine, Washington University, St. Louis, Missouri, United States of America; 5 Behavioral and Urban Health Program, Behavioral Health and Criminal Justice Division, Research Triangle Institute (RTI) International, Research Triangle Park, North Carolina, United States of America; 6 Fellow Program and Behavioral Health and Criminal Justice Division, RTI International, Research Triangle Park, North Carolina, United States of America; 7 Genometrics Section, Computational and Statistical Genomics Branch, National Human Genome Research Institute, National Institutes of Health, Baltimore, Maryland, United States of America; National Taiwan University, TAIWAN

## Abstract

Imputation, the process of inferring genotypes for untyped variants, is used to identify and refine genetic association findings. Inaccuracies in imputed data can distort the observed association between variants and a disease. Many statistics are used to assess accuracy; some compare imputed to genotyped data and others are calculated without reference to true genotypes. Prior work has shown that the Imputation Quality Score (IQS), which is based on Cohen’s kappa statistic and compares imputed genotype probabilities to true genotypes, appropriately adjusts for chance agreement; however, it is not commonly used. To identify differences in accuracy assessment, we compared IQS with concordance rate, squared correlation, and accuracy measures built into imputation programs. Genotypes from the 1000 Genomes reference populations (AFR N = 246 and EUR N = 379) were masked to match the typed single nucleotide polymorphism (SNP) coverage of several SNP arrays and were imputed with BEAGLE 3.3.2 and IMPUTE2 in regions associated with smoking behaviors. Additional masking and imputation was conducted for sequenced subjects from the Collaborative Genetic Study of Nicotine Dependence and the Genetic Study of Nicotine Dependence in African Americans (N = 1,481 African Americans and N = 1,480 European Americans). Our results offer further evidence that concordance rate inflates accuracy estimates, particularly for rare and low frequency variants. For common variants, squared correlation, BEAGLE R^2^, IMPUTE2 INFO, and IQS produce similar assessments of imputation accuracy. However, for rare and low frequency variants, compared to IQS, the other statistics tend to be more liberal in their assessment of accuracy. IQS is important to consider when evaluating imputation accuracy, particularly for rare and low frequency variants.

## Introduction

In genomic analyses high-quality data are crucial to accurate statistical inferences. Data accuracy can typically be assessed by different methods and measures.

Genetic imputation provides an informative scenario for examining how the use of different accuracy measures can influence the assessment of accuracy. Genotype imputation is a valuable tool in association studies and meta-analyses. This process infers “in silico” genotypes for untyped variants in a study sample by matching genotyped variants in the study to corresponding haplotypes in a comprehensively genotyped reference panel [1–8]. Therefore, imputation accuracy is influenced by haplotype frequencies in the reference panel [9–10] and the typed single nucleotide polymorphism (SNP) coverage of the study sample [11–12]. Once untyped variants are inferred, statistics that measure imputation accuracy are calculated to identify poorly imputed SNPs.

Imputation accuracy statistics can be classified into two types: (1) statistics that compare imputed to genotyped data and (2) statistics produced without reference to true genotypes. Concordance rate, squared correlation, and Imputation Quality Score (IQS) [13] are examples of the first type. Because imputed SNPs usually do not have genotyped data for comparison, statistics of the second type are usually provided by imputation programs and are commonly relied upon in practice. However, a direct comparison of imputed and genotyped data can be made possible by masking a percentage of variants that were genotyped in the study sample [9, 14–15].

Lin et al (2010) introduced IQS, which is based on Cohen’s kappa statistic for agreement [13]. Because of chance agreement, concordance rate, i.e. the proportion of agreement, can lead to incorrect assessments of accuracy for rare and low frequency variants. IQS adjusts for chance agreement [13]. Furthermore, Lin et al. (2010) used simulated data to show that requiring an IQS threshold > 0.9 removed all false positive association signals, while concordance rate > 0.99 still resulted in many false positives. Despite this evidence, IQS is not widely used in accuracy assessment.

This work builds upon previous studies by comparing IQS with commonly used accuracy measures—concordance rate, squared correlation, and built-in accuracy statistics—with the goal of identifying situations in which the choice of accuracy measure leads to differing assessments of accuracy. We compared imputed and genotyped data via masking, and used African-ancestry and European-ancestry populations to evaluate imputation accuracy in genomic regions associated with nicotine dependence and smoking behavior, some of which have also been implicated in lung cancer and chronic obstructive pulmonary disease (COPD).

## Methods

We examined differences and similarities in accuracy assessment as measured by IQS, squared correlation, concordance rate and built-in accuracy statistics using: (1) 1000 Genomes as the sample and the reference, and (2) data from nicotine dependence studies as the sample and 1000 Genomes as the reference. Below we describe both approaches, beginning with analyses involving 1000 Genomes as the sample and the reference.

### Masking and Imputation using 1000 Genomes Data

Because IQS adjusts for chance agreement [13], we used IQS as a benchmark for accuracy estimation. Calculating IQS, concordance rate, and squared correlation requires genotyped data for comparison with imputed data. We created a study sample for imputation by masking genotypes in the reference panel to mimic the typed SNP coverage of commercially available SNP arrays (Affymetrix—Affy 500 and Affy 6 as well as Illumina—Duo, Omni, and Quad matched by genomic position using Build 37.3/hg19). We used 1000 Genomes African (AFR) and European (EUR) continental reference panels with 246 and 379 individuals respectively (S1 Table) [16]. All data analyzed here are de-identified, publicly available data from the 1000 Genomes (1000G) project, which provides these data as a resource for the scientific community. Participants provided informed consent to the 1000G Project for broad use and broad data release in databases [16–17]. We also have Washington University Human Research Protection Office approval for analyses of de-identified data.

**Fig 1:**
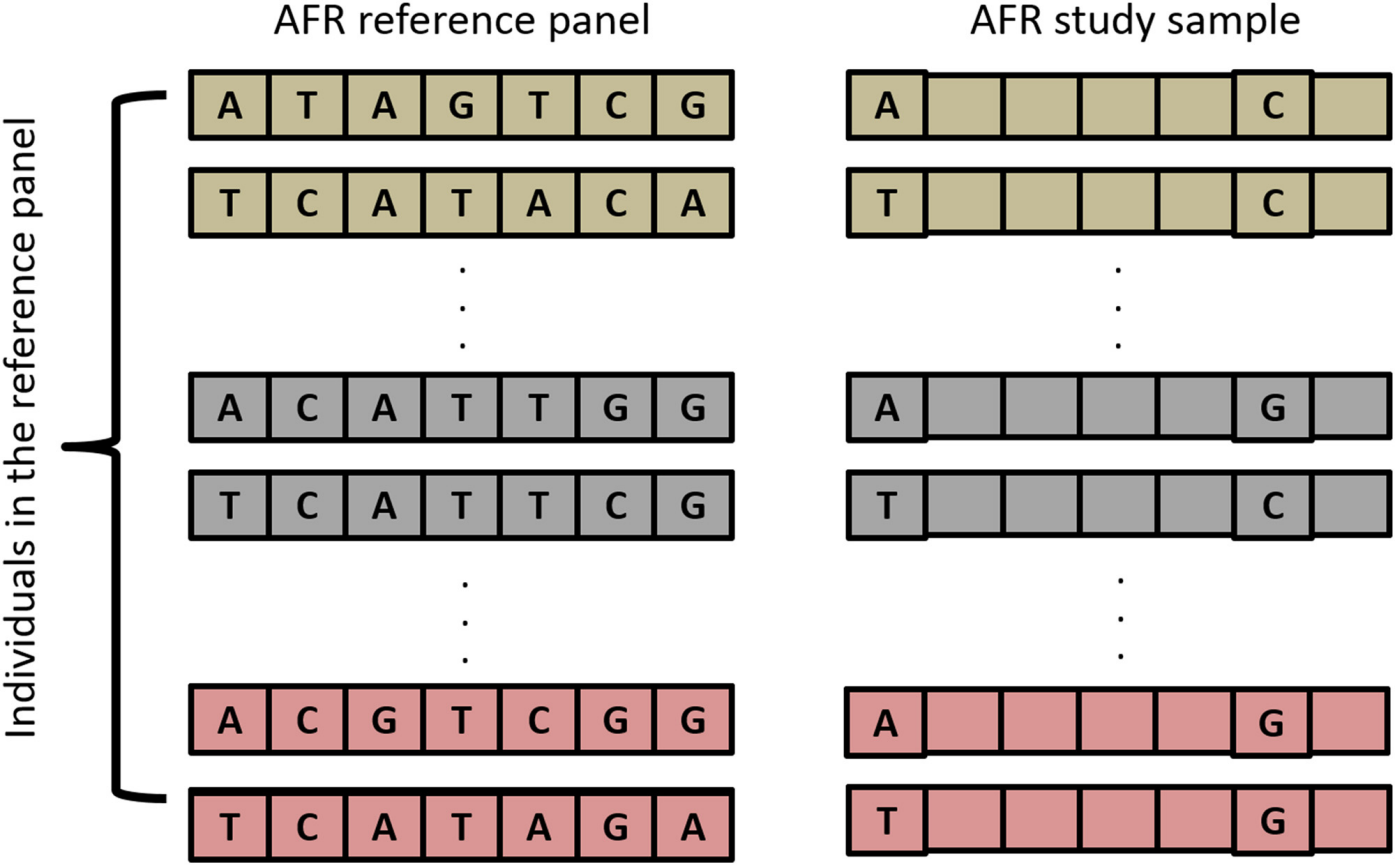
General process for creating the study sample for imputation. The reference panel was masked to mimic a commercial SNP array, resulting in a study sample which contains the same individuals as the reference panel.

The process of creating the study sample is described in Fig 1 and the numbers of typed variants are presented in S2 Table. Fig 1 illustrates several key characteristics of our masking approach. The reference panel individuals were the same as the study sample individuals. Our approach is expected to give an upper bound on accuracy because of the ideal match between the reference panel and study sample; the “correct” haplotype for each individual being imputed is present in the reference. Using population-specific reference panels (AFR and EUR) rather than a cosmopolitan reference panel maximizes the matching between the reference panel and study sample. Also, this design allowed us to compare accuracy estimates for variants not found on a SNP array. This sample data set was then imputed and the results were used to calculate accuracy statistics.

### Imputation Programs

BEAGLE (version 3.3.2) [2, 8] and IMPUTE2 [1, 4–5] were used to obtain imputed genotype probabilities. We obtained the BEAGLE R^2^ and IMPUTE2 INFO accuracy measures for each SNP; neither of these makes use of true genotypes. The BEAGLE R^2^ and IMPUTE2 INFO accuracy measures are well established [3, 15]. BEAGLE R^2^ approximates the squared correlation between the most likely genotype and the true unobserved allele dosage [2, 8]. IMPUTE2 INFO considers allele frequency as well as the observed and expected allele dosage [15]. We include their formulas for completeness, in Eqs 1 and 2, Here *g*_*n*_ represents the observed dosage, *e*_*n*_ represents the expected allele dosage, and $\hat{\theta }$ represents the sample allele frequency for sample n at a particular SNP, where n ranges from 1 to N, the total number of individuals and 0 < $\hat{\theta }$ <1. Additionally, *z*_*n*_ represents the genotype with the highest posterior probability from imputation, i.e. 0, 1, or 2 corresponding to the number of copies of the coded allele. Finally, *f*_*n*_ = *p*_*n1*_ + 4*p*_*n2*_ where *p*_*nk*_ represents the imputed probability of the genotypic class k (0, 1, and 2) corresponding to the nth sample.

$$\;BEAGLE\;R^{2}=\;\frac{{\left[\sum_{n=1}^{N}\;g_{n}e_{n}-\;\left(1/N\right)\left(\sum_{n=1}^{N}\;g_{n}\sum_{n=1}^{N}\;e_{n}\right)\right]}^{2}}{\left[\sum_{n=1}^{N}f_{n}-\left(1/N\right){\left(\sum_{n=1}^{N}e_{n}\right)}^{2}\right]\left[\sum_{n=1}^{N}z_{n}-\;\left(1/N\right){\left(\sum_{n=1}^{N}z_{n}\right)}^{2}\right]}$$ (1)

$$IMPUTE2\;INFO=\;1-\;\frac{\sum_{n=1}^{N}\left(f_{n}-\;e_{n}^{2}\right)}{2N\hat{\theta }\left(1-\hat{\theta }\right)}$$ (2)

Imputed probabilities produced by BEAGLE and the corresponding accuracy statistics showed variability, so we focus on these results. Analyses using IMPUTE2 were less informative in this matched sample-reference setting; this program appears to identify the matching individual in the reference and assign imputed data accordingly. The result was highly accurate imputation in this special context. Since we aim to compare concordance rate, squared correlation, and IQS in efforts to identify scenarios where these statistics produce similar or divergent conclusions regarding accuracy estimation, the variation produced by using BEAGLE for imputation allows us to address our question of interest.

### Statistics that Compare Genotyped and Imputed Data

The imputed genotype probabilities produced by BEAGLE and IMPUTE2 were used to calculate concordance rate, squared correlation and IQS. These imputed genotype probabilities, one for each genotype class (e.g. AA, AB, or BB), are transformed to dosage values by multiplying by 0, 1 or 2 for each genotypic class. IQS is calculated from genotype probabilities while squared correlation uses dosage values. Note that a specific dosage value can correspond to multiple genotypic probabilities, but only one dosage value can result from a specific set of genotypic probabilities. Although the most likely (best guess) genotype for each variant can be used to calculate these statistics, it is not recommended because the discrete classification of each individual’s genotype does not consider the probabilistic nature of imputation [18].

The incorporation of the genotypic classes into the IQS calculation is represented in Table 1, where each cell is the sum of the genotype probabilities for each genotyped and imputed genotypic class combination. The IQS calculation is demonstrated in Eq 3. IQS considers both the observed proportion of agreement (concordance rate or P_o_ shown in Eq 4) as well as chance agreement (P_c_ in Eq 5). Concordance rate (P_o_) is the sum of probabilities for each matching genotypic class divided by the total sum of all genotype probabilities. Chance agreement is evaluated as the sum of the products of the marginal frequencies. An IQS score of one indicates that the data matched perfectly, while a negative IQS score indicates that the SNP was imputed worse than expected by chance [13]. Mathematically, the value of IQS will always be less than or equal to the value of concordance rate: P_o_P_c_ ≤ P_c_, so P_o_−P_c_ ≤ P_o_-P_o_P_c_, hence (P_o_-P_c_)/(1-P_c_) ≤ (P_o_-P_o_P_c_)/(1-P_c_), which says that IQS ≤ P_o_. Some statistics can be confounded with Hardy-Weinberg equilibrium (HWE) if they assume HWE to calculate "expected" genotype counts [19]. IQS avoids this concern since it uses imputed and experimentally determined genotypes.

**Table 1:** Calculating concordance (P_0_) and IQS from imputed genotype probabilities and actual genotypes. The table was created by summing over probabilities for all N individuals (n = 1 to N) in each cell with p_ij_n_ representing the probability that the nth individual has the imputed genotype i and actual genotype j, where 1 corresponds to AA, 2 corresponds to AB, and 3 corresponds to BB. N_1_ = number of individuals with AA actual genotype, N_2_ = number of individuals with AB actual genotype, N_3_ = number of individuals with BB actual genotype, and N = number of total individuals.

			Actual		
		AA	AB	BB	Total
	AA	$\overset{N}{\underset{n=1}{\sum }}\;{\text{p}}_{11\_\text{n}}$	$\overset{N}{\underset{n=1}{\sum }}\;{\text{p}}_{12\_\text{n}}$	$\overset{N}{\underset{n=1}{\sum \;}}{\text{p}}_{13\_\text{n}}$	$\;\overset{3}{\underset{j=1}{\sum }}\overset{N}{\underset{n=1}{\sum }}\;{\text{p}}_{1\text{j}\_\text{n}}$
	AB	$\overset{N}{\underset{n=1}{\sum }}\;{\text{p}}_{21\_\text{n}}$	$\overset{N}{\underset{n=1}{\sum }}\;{\text{p}}_{22\_\text{n}}$	$\overset{N}{\underset{n=1}{\sum }}\;{\text{p}}_{23\_\text{n}}$	$\overset{3}{\underset{j=1}{\sum }}\overset{N}{\underset{n=1}{\sum }}\;{\text{p}}_{2\text{j}\_\text{n}}$
Imputed	BB	$\overset{N}{\underset{n=1}{\sum }}\;{\text{p}}_{31\_\text{n}}$	$\overset{N}{\underset{n=1}{\sum }}\;{\text{p}}_{32\_\text{n}}\;$	$\overset{N}{\underset{n=1}{\sum }}\;{\text{p}}_{33\_\text{n}}$	$\overset{3}{\underset{j=1}{\sum }}\overset{N}{\underset{n=1}{\sum }}\;{\text{p}}_{3\text{j}\_\text{n}}$
	Total	$\overset{3}{\underset{i=1}{\sum }}\overset{N}{\underset{n=1}{\sum \;}}{\text{p}}_{\text{i}1\_\text{n}}=N\text{1}$	$\overset{3}{\underset{i=1}{\sum }}\overset{N}{\underset{n=1}{\sum }}\;{\text{p}}_{\text{i}2\_\text{n}}=N\text{2}$	$\overset{3}{\underset{i=1}{\sum }}\overset{N}{\underset{n=1}{\sum }}\;{\text{p}}_{\text{i}3\_\text{n}}=N\text{3}$	N

$$\text{IQS }=\frac{Po-Pc}{1-Pc}$$ (3)

$$\;Po=\frac{\sum_{n=1}^{N}\;{\text{p}}_{11_{\text{n}}}+\sum_{n=1}^{N}\;{\text{p}}_{22_{\text{n}}}+\;\sum_{n=1}^{N}\;{\text{p}}_{33_{\text{n}}}\;}{\text{N}}$$ (4)

$$Pc=\frac{\text{N1}*\sum_{j=1}^{3}{\;\sum }_{n=1}^{N}\;{\text{p}}_{1\text{j}\_\text{n}}+\text{ N2}*\sum_{j=1}^{3}\;\sum_{n=1}^{N}\;{\text{p}}_{2\text{j}\_\text{n}}+\text{N3}*\sum_{j=1}^{3}\;\sum_{n=1}^{N}\;{\text{p}}_{3\text{j}\_\text{n}}}{{\text{N}}^{2}}$$ (5)

Squared correlation is the square of the Pearson correlation coefficient between the imputed and genotyped dosage for each SNP. This is calculated using Eqs 6–11 where x_i_ and y_j_ are the imputed and genotyped dosage values for the nth sample respectively. It represents the proportion of the variability in the imputed data that can be explained by the least squared regression model.

$${\text{R}}^{2}\;=1-\;\frac{SSE}{SS_{yy}}$$ (6)

$${\text{SS}}_{\text{yy}}=\sum_{n=1}^{N}{\left(y_{i}-\bar{y}\right)}^{2}$$ (7)

$$\text{SSE }=SS_{yy}-\hat{\beta_{n}}\left(SS_{xy}\right)$$ (8)

$$\;{\text{SS}}_{\text{xy}}=\;\sum_{n=1}^{N}\left(y_{i}-\;\bar{y}\right)\left(x_{j}-\;\bar{x}\right)$$ (9)

$$\hat{\beta_{\text{n}}}\;=\frac{SS_{xy}}{SS_{xx}}$$ (10)

$${\text{SS}}_{\text{xx}}=\;\sum_{n=1}^{N}{\left(x_{j}-\bar{x}\right)}^{2}$$ (11)

### Evaluating Accuracy across MAF and LD

Imputation accuracy is influenced by a variant’s minor allele frequency (MAF) and linkage disequilibrium (LD) with genotyped variants (measured by pairwise squared correlation r^2^). We examined imputation accuracy in relation to these properties. The MAFs used here were based on the allele frequencies found in the genotyped data. We will use the terminology “rare” to denote variants with MAF ≤ 1%; and “low frequency” to refer to variants with 1% < MAF ≤ 5%. For each imputed SNP, the genotyped SNP in the region with the highest LD was used to define the maximum r^2^_LD_ with a genotyped SNP (denoted by max r^2^_LD_). PLINK was used to generate the LD values [20]. Bins for maximum r^2^_LD_ and MAF were defined in 0.01 increments [13]. For each bin, the mean and one standard deviation of the values produced by each accuracy statistic were calculated.

### Examining Regions Associated with Nicotine Dependence

We examined the imputation accuracy of two genomic regions known to be associated with nicotine dependence and smoking behavior. These regions were the nicotinic receptor subunit gene clusters on chromosome 15 (*CHRNA5*-*CHRNA3*-*CHRNB4*) and chromosome 8 (*CHRNB3*-*CHRNA6*) [21–26]. These signals were identified through genome-wide association studies (GWAS) and meta-analyses for smoking behavior, with the chromosome 15 region being the most significantly associated. We imputed 3Mb on each chromosome: 2Mb regions used for analysis plus two 500Kb flanking buffer regions according to Build 37.3/hg19. We focused our analyses on polymorphic variants with dbSNP identifiers in each 2MB region.

### Masking and Imputation in a Real Data Application using a Nicotine Dependence Sample

A comparison of accuracy statistics was also conducted using nicotine dependence data as the study samples (N = 1,481 African Americans and N = 1,480 European Americans who were sequenced) and 1000 Genomes as the reference. The study sample was masked and imputed separately by race. This analysis provided a more conventional imputation scenario for comparison with the patterns found in the 1000 Genomes analyses.

The sequenced subjects in this applied analysis were from the Collaborative Genetic Study of Nicotine Dependence (COGEND) and the Genetic Study of Nicotine Dependence in African Americans (AAND). These studies are cross-sectional and contain extensive smoking behavior phenotypes in African Americans and European Americans [21]. These individuals were between the ages of 25–44 years old and were assessed for dependence as measured by the Fagerstrom Test for Nicotine Dependence (FTND) and cigarettes-per-day (CPD) [27]. The study protocol was approved by the appropriate Institutional Review Boards and written informed consent was obtained from all subjects.

Center for Inherited Disease Research (CIDR) performed next-generation targeted sequencing on genomic regions previously associated with smoking behaviors, using COGEND and AAND DNA samples derived from blood. Genotypic data that passed initial quality control at CIDR were released to the Quality Assurance/Quality Control analysis team at the University of Washington Genetics Coordinating Center. These data had mean on-target coverage of 180X with more than 96% of on-target bases containing a depth greater than 20X. A total of 1,481 African Americans and 1,480 European Americans were used in the analysis.

These sequencing data were masked to match the typed SNP coverage of the Omni 2.5 SNP array in a 500kb region on chromosome 15. The cosmopolitan reference panel, composed of individuals from a variety of ancestries, was used for imputation since it has been shown to produce the best accuracy estimates [9]. The imputation was performed using BEAGLE and IMPUTE2 to evaluate whether observed trends in accuracy were consistent across imputation programs. The imputed probabilities were compared to the masked sequencing data and accuracy statistics were calculated. We focused our analyses on polymorphic variants.

## Results

**Fig 2:**
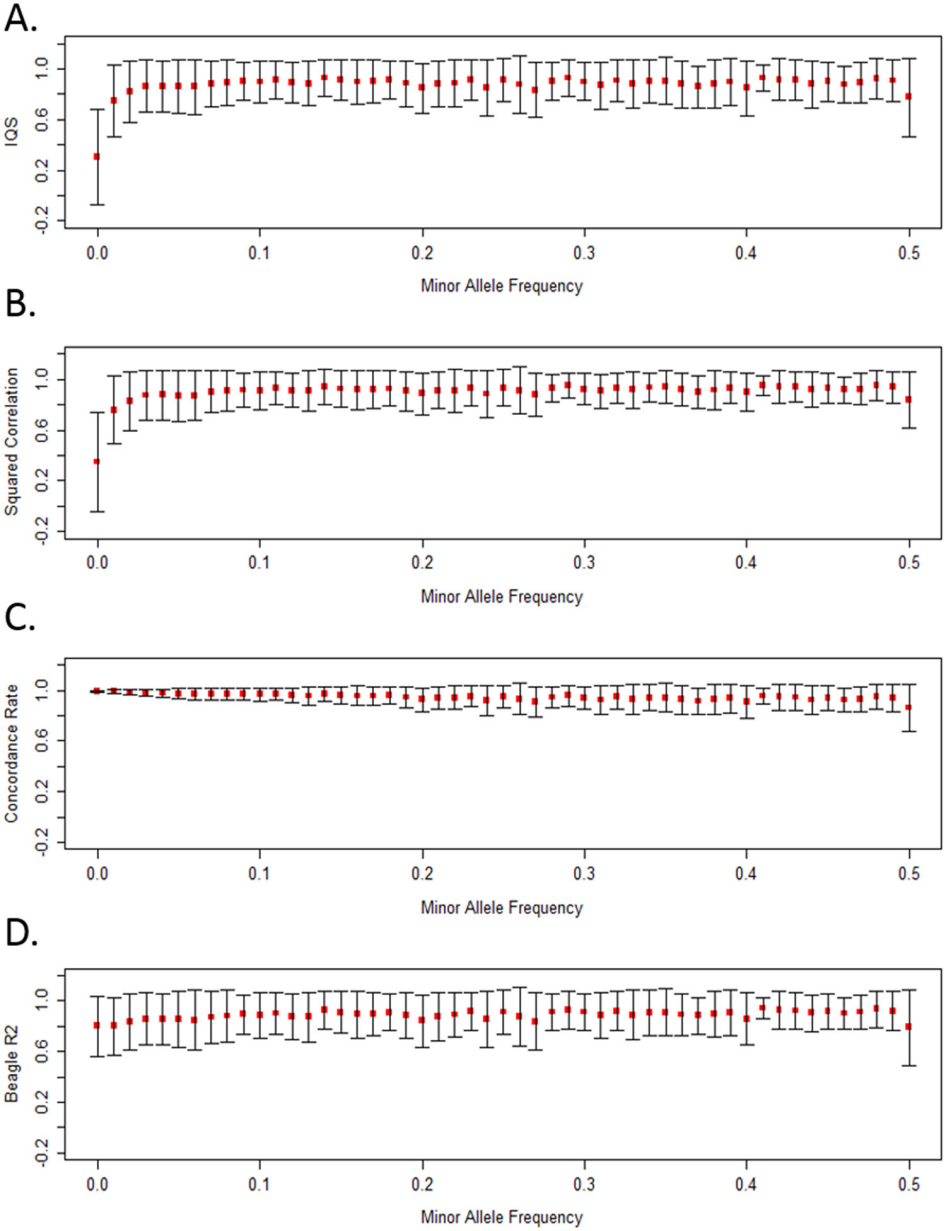
IQS, squared correlation, concordance rate, and BEAGLE R^2^ are shown in MAF bins. Mean accuracy of SNPs in each MAF bin (defined by 0.01 increments with N = 13,442 variants total) is denoted by the red dots and the bars indicate one standard deviation (above and below the mean). These results are produced by using the 1000 Genomes AFR reference population as the study sample with Omni 2.5M typed coverage on chromosome 15.

**Fig 3:**
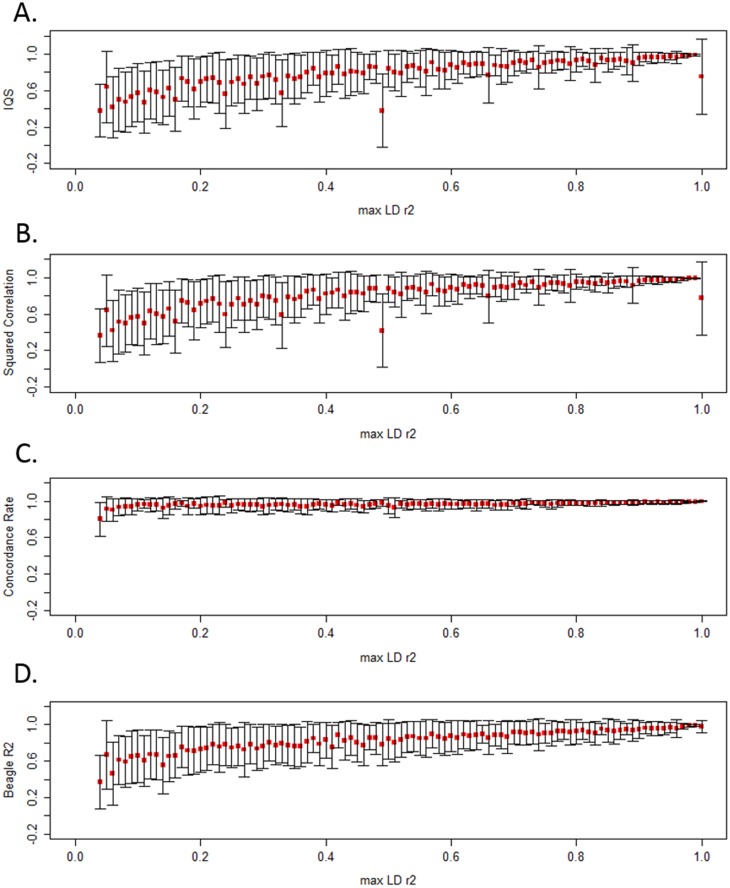
IQS, squared correlation, concordance rate, and BEAGLE R^2^ are shown in max r^2^_LD_ bins. Mean accuracy of SNPs in each MAF bin (defined by 0.01 increments with N = 13,442 variants total) is denoted by the red dots and the bars indicate one standard deviation (above and below the mean). These results were produced by using the 1000 Genomes AFR reference population as the study sample with Omni 2.5M typed coverage on chromosome 15.

**Fig 4:**
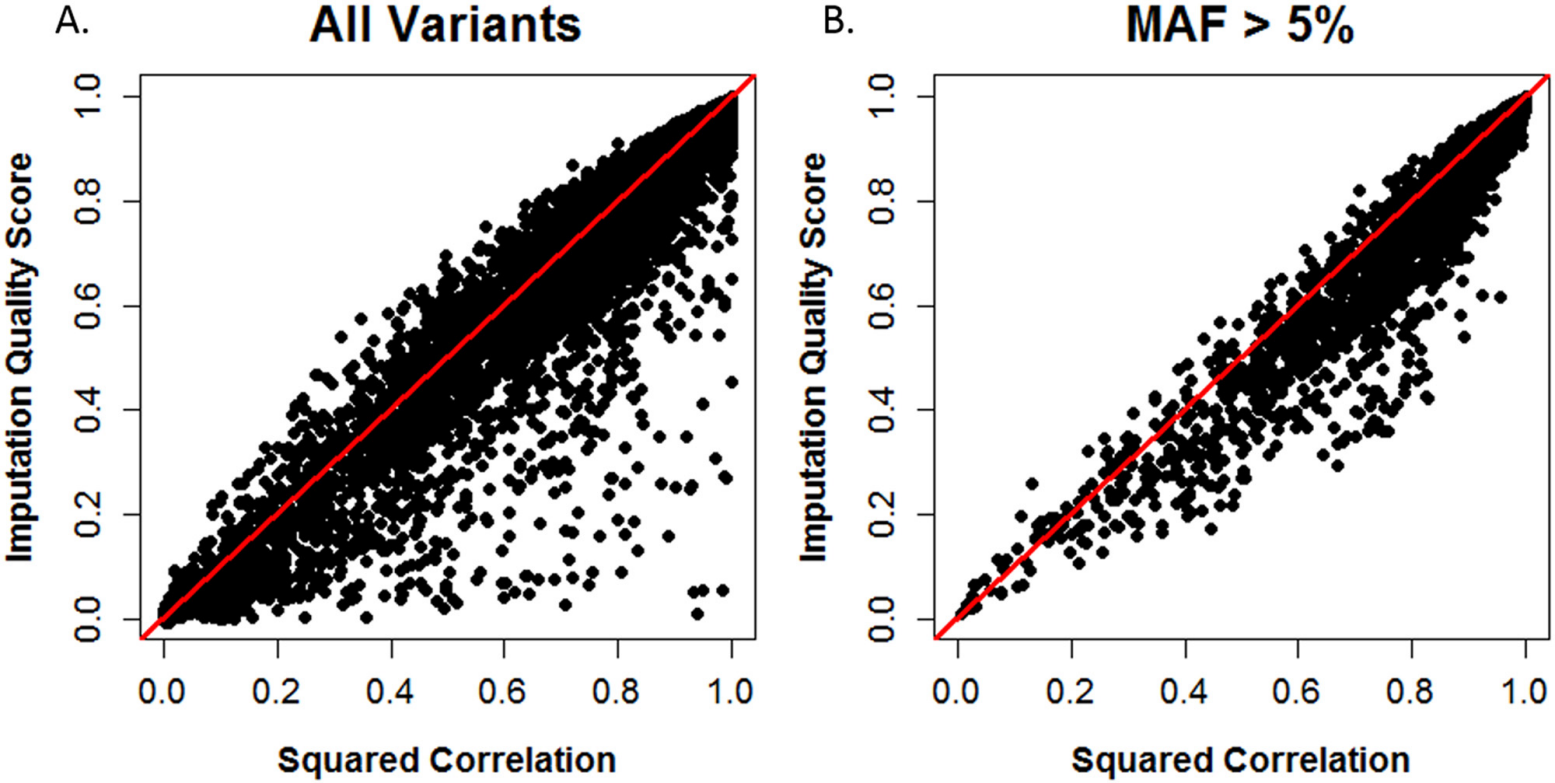
Scatterplots of squared correlation and IQS. Data for all 13,442 variants are displayed in panel A, while the results for variants with MAF>5% (N = 6,480) are found in panel B. The line y = x is denoted in red.

**Fig 5:**
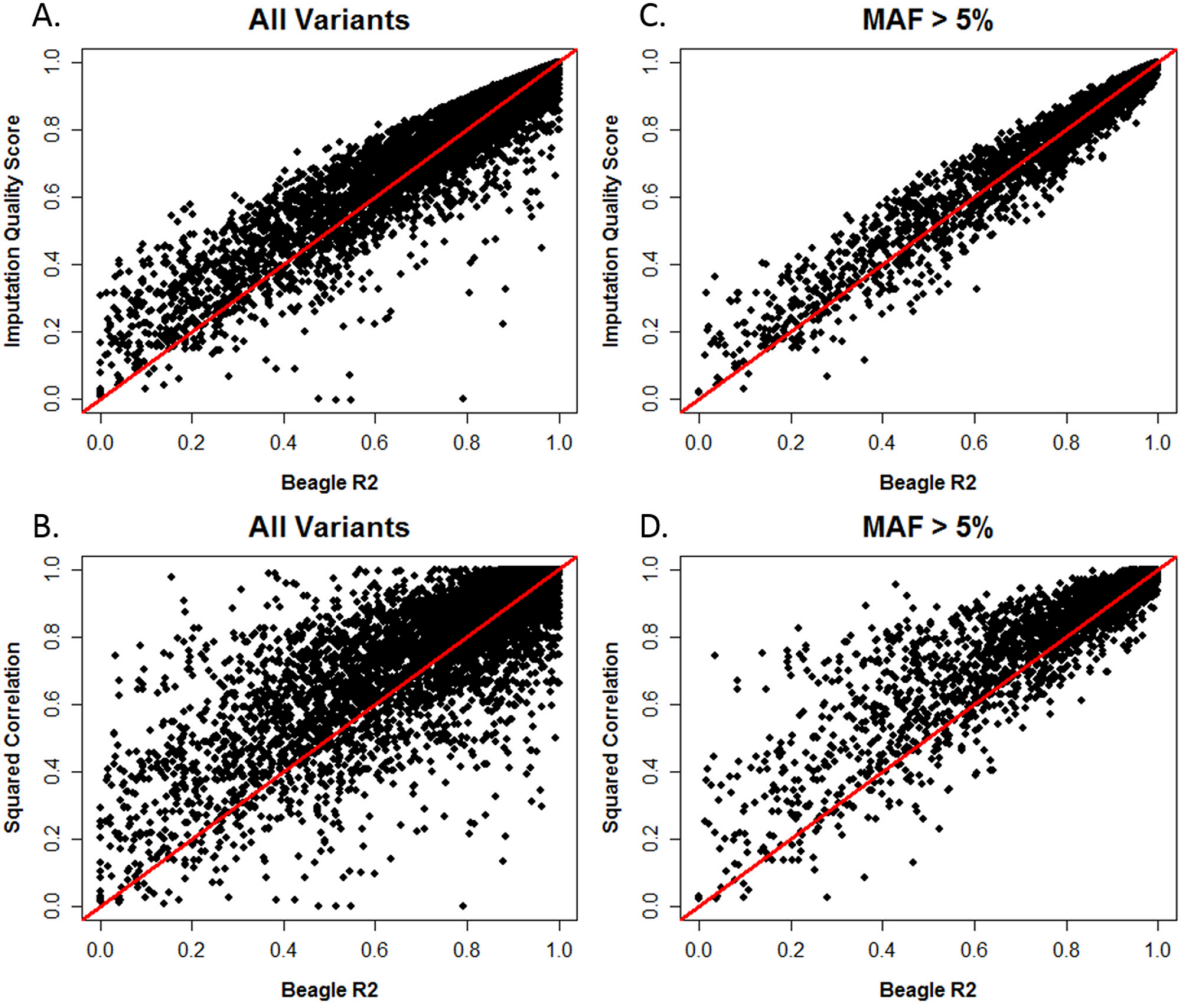
Scatterplots of IQS, squared correlation, and BEAGLE R^2^. Panels A and B display all 13,442 variants, and panels C and D display variants with MAF>5% (N = 6,480). The line y = x is denoted in red.

We compared IQS with squared correlation, concordance rate, and BEAGLE R^2^ to examine changes in accuracy assessment using 1000 Genomes as the study sample in Figs 2–5. IQS is our benchmark because it adjusts for chance agreement, in contrast to concordance rate which inflates assessments of accuracy [13]. We focus here on the results for the AFR reference population using Omni 2.5M typed coverage on chromosome 15 (13,442 imputed SNPs). We emphasize Omni 2.5 because it has the greatest genotype SNP coverage in the region (S2 Table).

### Results for 1000 Genomes Imputation with Matching Reference

Results produced using BEAGLE and the AFR reference population are shown. Results for different chromosomal regions and populations were similar and are shown in S6–S8 Figs.

To help interpret results that are displayed by MAF and max r^2^_LD_ bin, S1 Fig. shows the number of imputed variants in each MAF bin in panel A and max r^2^_LD_ bin in panel B. This figure indicates that most of the imputed variants were rare and low frequency variants. There were 6,480 (48.21%) rare and low frequency rsID SNPs in the AFR population. The bins ranged in size from 7 variants (0.49 ≥ MAF < 0.50) to 2,371 variants (0.01 ≥ MAF < 0.02).

### Concordance Rate and BEAGLE R^2^ Inflate Assessments of Accuracy for Rare Variants

Results show that the choice of statistic is important when examining the imputation accuracy of rare and low frequency variants. Fig 2 displays the mean accuracy and one standard deviation in each MAF bin, after imputing from Omni 2.5M coverage. IQS (Panel A) and squared correlation (Panel B) produced similar means and standard deviations in each bin, though this does not necessarily represent similarity of values for particular SNPs. For rare and low frequency variants, both concordance rate (Panel C) and BEAGLE R^2^ (Panel D) produce inflated assessments of accuracy. The higher concordance rate and BEAGLE R^2^ values could mislead a researcher into assuming that these variants were imputed well, and that accuracy is best measured using concordance rate and BEAGLE R^2^. IQS and squared correlation also show low accuracy for rare variants using other SNP array coverages (S2 Fig).

A MAF bin can have a wide range in accuracy values. Fig 2 shows variability within MAF bins across all MAF values. Standard deviations for IQS, squared correlation and BEAGLE R^2^ can be sizeable for both rare and common variants (panels A, B and D); concordance rate does not reflect this as it classifies most variants as well imputed (panel C).

### Rare and Low Frequency Variants can be Well Tagged but Poorly Imputed

We examined max r^2^_LD_, the maximum LD r^2^ between imputed and genotyped SNPs, to understand the relationship between typed SNP coverage and imputation accuracy as measured by these accuracy statistics. Fig 3 displays the mean accuracy and one standard deviation in each max r^2^_LD_ bin, after imputing from Omni 2.5M coverage, additional arrays are in S3 Fig. Mean accuracy tends to increase with increasing max r^2^_LD_, as expected. For low to moderate max r^2^_LD_, we observed substantial variability in IQS as well as squared correlation and BEAGLE R^2^ values; however, at high max r^2^_LD_, the variability decreases. IQS and squared correlation show a surprisingly wide standard deviation for variants in the highest max r^2^_LD_ bin (0.99 < max r^2^_LD_ ≤ 1) as well as the max r^2^_LD_ bin 0.5 < max r^2^_LD_ ≤ 0.51. Upon investigation, we found that the variability was due to rare variants: after limiting to SNPs with MAF > 5%, these standard deviations were comparable to those of the other bins, S4 Fig. This pattern suggests that even rare variants that are well tagged (as measured by max r^2^_LD_) can be poorly imputed.

### Concordance Classifies Most Variants as Well Imputed

Concordance differs from IQS, squared correlation, and BEAGLE R^2^ in that it indiscriminately classifies most variants as well imputed, across MAF (Fig 2) and r^2^_LD_ bins (Fig 3). The results in Figs 2 and 3 support prior concerns regarding concordance rate [13] and led us to focus the rest of our evaluation on IQS, squared correlation, and BEAGLE R^2^.

### For Rare Variants, IQS and Squared Correlation Produce Different Assessments of Accuracy

Although squared correlation and IQS appeared similar overall in their assessment of imputation accuracy when examined using means and standard deviations by bin (Figs 2 and 3), further investigation showed that on an individual SNP level, these statistics produce divergent assessments of accuracy for rare and low frequency variants. We compared accuracy estimates produced by IQS and squared correlation in Fig 4 for each SNP. Panel A shows results for all variants, and panel B displays results for variants with MAF > 5%. A comparison of these panels is useful to identify divergent trends for common variants versus rare and low-frequency variants. For most SNPs, IQS and squared correlation produced similar assessments of accuracy as seen by the many observations on and near the y = x line in panels A and B. This is consistent with the accuracy patterns observed for IQS and squared correlation in Figs 2 and 3. However, discrepancies in accuracy assessment do occur, with squared correlation generally being more liberal in assigning high accuracy compared to IQS. This is indicated by the sparseness of observations above the y = x line in panels A and B. The points below the y = x line indicate SNPs for which squared correlation values were higher than IQS. Panel B shows that widely discrepant values for IQS and squared correlation are attributable to rare and low frequency SNPs: filtering out SNPs with MAF ≤ 5% removes the widely discrepant observations.

To further examine trends in the discrepancies between these statistics, we subtracted squared correlation from IQS for each variant and displayed this result across all MAF values in S5 Fig. Thus negative differences denote that squared correlation was greater than IQS (i.e. squared correlation more liberal) while positive differences indicate that IQS was greater than squared correlation. Large discrepancies occur over all MAF values with squared correlation tending to be higher than IQS, especially for SNPs with higher MAFs.

### For Common Variants, IQS and BEAGLE R^2^ Provide Similar Assessments of Accuracy

For common variants, BEAGLE R^2^ produces a similar assessment of imputation accuracy as IQS, but BEAGLE R^2^ can differ dramatically from squared correlation. In Fig 5, we compared BEAGLE R^2^ to IQS (panels A and C) and squared correlation to BEAGLE R^2^ (panels B and D). For many variants, squared correlation and BEAGLE R^2^ differ in accuracy assessment as seen by the variants above the y = x line in panel B. Although most of these variants are rare, there are still many common variants for which this trend is true (panel D). Large differences between IQS and BEAGLE R^2^ occur mostly when rare variants are examined.

### Results are Similar in Different Genomic Regions and Populations

Figs 2–5 displayed results for the AFR reference population and Omni 2.5M typed coverage in the chromosome 15 region. Results similar to those described above were also observed using the AFR reference on chromosome 8 (S6 Fig) as well as using the EUR reference panel for chromosomes 15 and 8 (S7 and S8 Figs respectively). In particular, low IQS values do occur for rare variants that have high squared correlation or high BEAGLE R^2^. The number of variants for each imputation subset can be found in S3 Table.

### Results are Consistent in Application to Nicotine Dependence Study Sample

**Fig 6:**
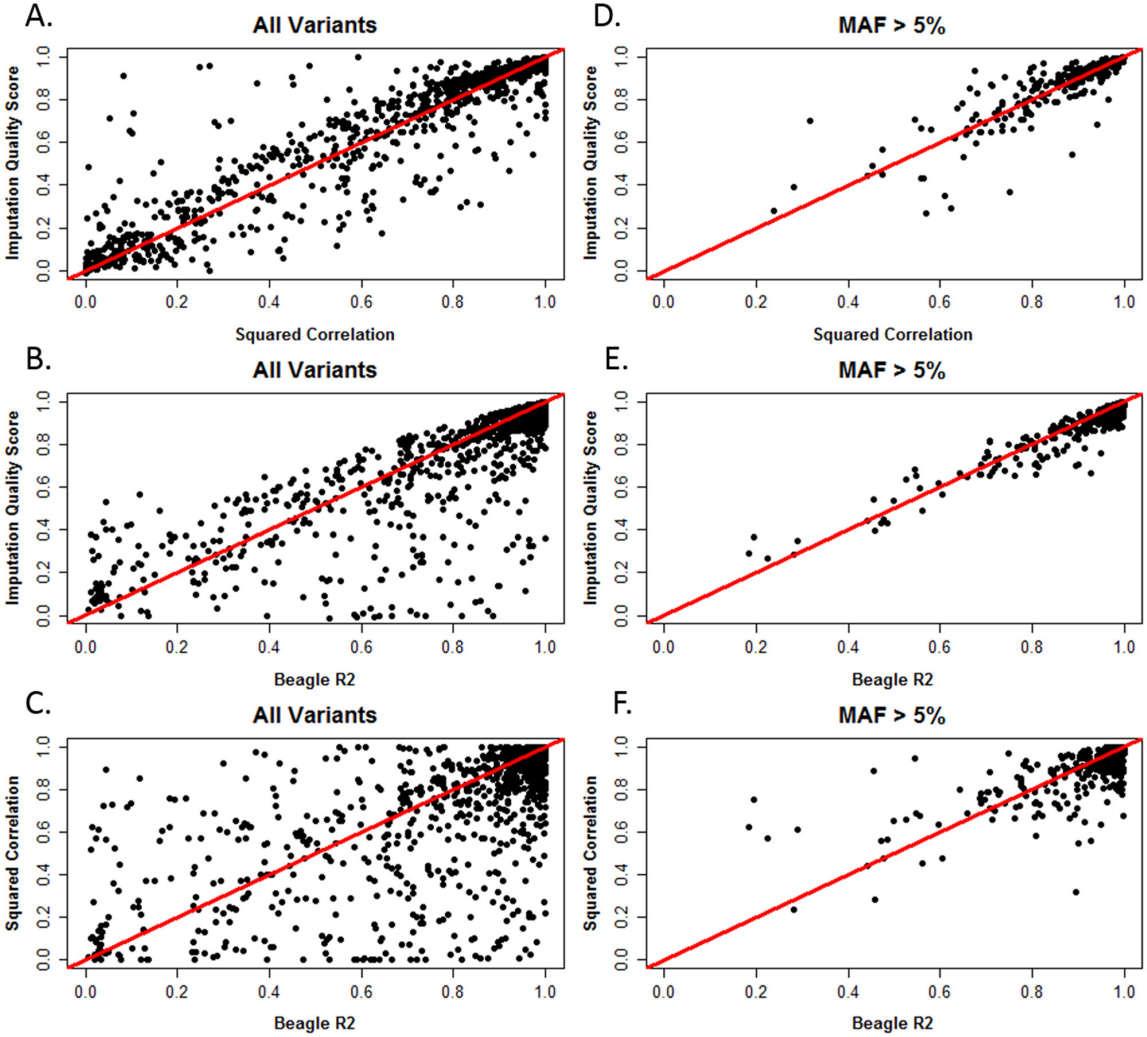
Scatterplots of IQS, squared correlation, and BEAGLE R^2^ using the cosmopolitan reference panel and the African American nicotine dependence study sample for chromosome 15. Data for all 1,545 variants are displayed in panel A, B, and C while the results for variants with MAF>5% (N = 631) are found in panel D, E, and F. These results were generated using Omni SNP coverage. The line y = x is denoted in red.

Fig 6 shows results produced using African American individuals from the nicotine dependence data as the study sample and a 1000 Genomes cosmopolitan reference panel imputed using BEAGLE. These data show discrepancies in accuracy assessment between statistics. If IQS and squared correlation are compared, squared correlation tends to be similar or higher (i.e. more liberal) than IQS. In the applied scenario, we observed some variants with high IQS and low squared correlation (Fig 6, panel A, upper left quadrant), which was not observed for the upper bound values from the 1000 Genomes analysis (Fig 4, panel A); however, these discrepancies are few, and mostly among rare and low frequency variants (see Fig 6, panel D). When comparing IQS to Beagle R^2^, the applied scenario showed IQS to be similar to or less than Beagle R^2^ (Fig 6, panel B), which recapitulates patterns seen in 1000 Genomes (Fig 5, panel A).

In European Americans, from the nicotine dependence data, we also observed these same patterns as in African Americans, with squared correlation’s more liberal assignment of accuracy as compared to IQS, S9 Fig. These results were also consistent using IMPUTE2 with African American and European American study samples, S10 and S11 Figs respectively. This confirms that these patterns are not limited to specific populations, chromosomes, or imputation programs.

## Discussion

Genotype imputation is used to improve the density of genomic coverage and increase power by combining datasets [28], in efforts to identify and refine genetic variants associated with disease. We investigated how assessment of imputation accuracy changes when concordance rate, squared correlation and BEAGLE R^2^ are compared to IQS, focusing on two genomic regions associated with smoking behavior.

Results showed that the choice of accuracy statistic matters for rare variants more than for common variants. This is important given that researchers are increasingly interested in imputing rare and low frequency variants [29–31]. While it has been recognized that rare variants are more difficult to impute accurately, our work here goes further by highlighting that choice of accuracy measure has an important role.

For common variants, squared correlation, IMPUTE2, and BEAGLE R^2^ produce similar assessments of imputation accuracy as compared to IQS. For rare and low frequency variants, we observed varying assessments of accuracy compared to IQS. Our results also showed that discrepancies between IQS and squared correlation are most likely to occur at rare and low frequency variants, where squared correlation is more liberal in assigning higher accuracy as compared to IQS. An evaluation of nicotine dependence samples also showed discrepancies between IQS and squared correlation. We recommend calculating IQS to confirm imputation accuracy, especially for rare or low frequency variants.

The variability observed within a MAF or max r^2^_LD_ bin is a reminder that not all variants that share the same MAF or max r^2^_LD_ value can be imputed with the same level of accuracy. This is consistent with the expectation that the inference of untyped variants depends on haplotype block structure and not simply the pairwise relationships between the genotyped and untyped variants. For rare variants, high LD with a genotyped SNP may not guarantee high imputation accuracy. Still, overall, a high max r^2^_LD_ usually implies high accuracy, as we observed increasing mean accuracy along with decreasing variability within max r^2^_LD_ bins as max r^2^_LD_ increases.

We applied this approach to genomic regions associated with our phenotype of interest, smoking behavior using an upper bound scenario and a nicotine dependence sample. Thus, one limitation is that rather than comprehensively examining the genome, we focused only on selected genomic regions. Furthermore we focused on certain populations (European and African ancestry). Nevertheless, different regions (on chromosome 8 and 15), different imputation programs, and different populations showed similar overall patterns, suggesting that our observations are relevant throughout the genome and across multiple populations.

In our masking process using only the 1000 Genomes reference data, the reference panel individuals were the same as the study sample individuals, and our masked SNPs are not limited to a SNP array, making our approach different from the two most common masking processes. One common masking method removes the genotypes for a portion of markers (e.g. 10%) found amongst the typed variants on a study sample SNP array. This method can provide accuracy comparisons only for SNPs on the array. Our approach is able to provide accuracy assessments for SNPs not on the array.

Another commonly used masking method is the “leave-one-out” masking of a comprehensively genotyped reference panel, in which one individual is imputed using the remaining reference panel members. Our study design differed from the leave-one-out method since all individuals in the reference panel and study sample were the same. Our approach was expected to give an upper bound on accuracy because of the ideal match between the reference and study sample; the “correct” genotype for each individual at each variant was present in the reference panel.

Our results provide further evidence that concordance rate inflates accuracy estimates particularly for rare and low frequency variants [13, 32]. These observations highlight a need to account for chance agreement not only when assessing imputation accuracy, but also more broadly in other situations for which concordance is traditionally used to assess accuracy, such as checking genotype agreement across duplicate samples [33–34]. Concordance rate will always produce a value greater than or equal to IQS due to their mathematical relationship (see Methods for proof).

IQS is important to consider, as it is designed to identify variants for which imputation accuracy is better than can be expected by chance; accordingly, other measures were generally more liberal in assigning high accuracy. Our analyses indicate that especially for rare and low frequency variants, IQS may be important to avoid overly liberal assessments of imputation quality. In practice, IQS can be computed by the leave-one-out method. Databases that provide per-SNP "imputability," such as that created by Duan et al. [35], would have increased usefulness if they included IQS values. As imputation methodology continues to develop and reference panels become more comprehensive, we expect that imputation will become increasingly accurate. However, it will be important to take chance agreement into account when assessing this accuracy, and IQS provides a means to do so.

## Supporting Information

S1 Fig Mean numbers of polymorphic variants in each MAF (panel A) and max r^2^_LD_ (panel B) bin. These results are for the AFR population on chromosome 15 (13,442 imputed SNPs).
(TIF)

S2 Fig Average accuracy of all SNPs according to 0.01 incremental MAF bins for each accuracy measure using several typed SNP array coverages. These results were produced by using the 1000 Genomes AFR reference populations as the study samples for chromosome 15.
(TIF)

S3 Fig Average accuracy of all SNPs in 0.01 incremental max r^2^_LD_ bins for each accuracy measure using several typed SNP array coverages. These results were produced by using the 1000 Genomes AFR reference population as the study sample for chromosome 15.
(TIF)

S4 Fig Accuracy scores produced by IQS, squared correlation, concordance rate and Beagle R^2^ for SNPs with MAF > 5% (N = 6,480 SNPs) in max r^2^_LD_ bins. Bins are defined by 0.01 increments. Mean accuracy is denoted by the red dots and the bars indicate one standard deviation (above and below the mean). These results were produced by using 1000 Genomes AFR reference population as the study sample with Omni 2.5M typed coverage on chromosome 15.
(TIF)

S5 Fig Relationship between squared correlation and IQS by MAF. Squared correlation was subtracted from IQS for variants on chromosome 15 in the 1000 Genomes AFR reference population (N = 13,442 variants) as the study sample. Negative values indicate that the squared correlation score was higher while the positive values indicate that the IQS value was higher. The red line indicates the line y = 0.
(TIF)

S6 Fig Scatterplots of IQS, squared correlation, and BEAGLE R^2^ using the 1000 Genomes AFR reference panel as the study sample for chromosome 8. Data for all 10,937 variants are displayed in panel A, B, and C while the results for variants with MAF>5% (N = 4,533) are found in panel D, E, and F. These results were generated using Omni SNP coverage. The line y = x is denoted in red.
(TIF)

S7 Fig Scatterplots of IQS, squared correlation, and BEAGLE R^2^ using the 1000 Genomes EUR reference panel as the study sample for chromosome 15. Data for all 9,401 variants are displayed in panel A, B, and C while the results for variants with MAF>5% (N = 4,627) are found in panel D, E, and F. These results were produced by using Omni SNP coverage. The line y = x is denoted in red.
(TIF)

S8 Fig Scatterplots of IQS, squared correlation, and BEAGLE R^2^ using the 1000 Genomes EUR reference panel as the study sample for chromosome 8. Data for all 7,401 variants are displayed in panel A, B, and C while the results for variants with MAF>5% (N = 1,903) are found in panel D, E, and F. These results were produced by using Omni SNP coverage. The line y = x is denoted in red.
(TIF)

S9 Fig Scatterplots of IQS, squared correlation, and BEAGLE R^2^ using the cosmopolitan reference panel and the European American nicotine dependence study sample for chromosome 15. Data for all 1,170 variants are displayed in panel A, B, and C while the results for variants with MAF>5% (N = 387) are found in panel D, E, and F. These results were produced by using Omni SNP coverage. The line y = x is denoted in red.
(TIF)

S10 Fig Scatterplots of IQS, squared correlation, and IMPUTE2 INFO using the cosmopolitan reference panel and the African American nicotine dependence study sample for chromosome 15. Data for all 1,878 variants are displayed in panel A, B, and C while the results for variants with MAF>5% (N = 475) are found in panel D, E, and F. These results were generated using Omni SNP coverage. The line y = x is denoted in red.
(TIF)

S11 Fig Scatterplots of IQS, squared correlation, and IMPUTE2 INFO using the cosmopolitan reference panel and the European American nicotine dependence study sample for chromosome 15. Data for all 1,253 variants are displayed in panel A, B, and C while the results for variants with MAF>5% (N = 259) are found in panel D, E, and F. These results were generated using Omni SNP coverage. The line y = x is denoted in red.
(TIF)

S1 Table Sub-populations in the BEAGLE and IMPUTE2 AFR and EUR reference panels. (PDF)

S2 Table Numbers of SNPs in the 1000 Genomes study samples. Study sample variants were those found on each commercially available SNP array for the 2 MB chromosomal regions of interest. Only variants with dbSNP identifiers are listed in the number of variants in the reference panel column.
(PDF)

S3 Table Polymorphic, imputed SNPs used in the comparison of accuracy measures. These variants were found in the 2 MB chromosomal regions of interest using 1000 Genomes as the study sample and were imputed using Omni 2.5 coverage.
(PDF)
