# Supplementary figures and images for: When Does Choice of Accuracy Measure Alter Imputation Accuracy Assessments?
Source: PLoS One. 2015 Oct 12;10(10):e0137601. doi: 10.1371/journal.pone.0137601 (PMC4601794; doi:10.1371/journal.pone.0137601)

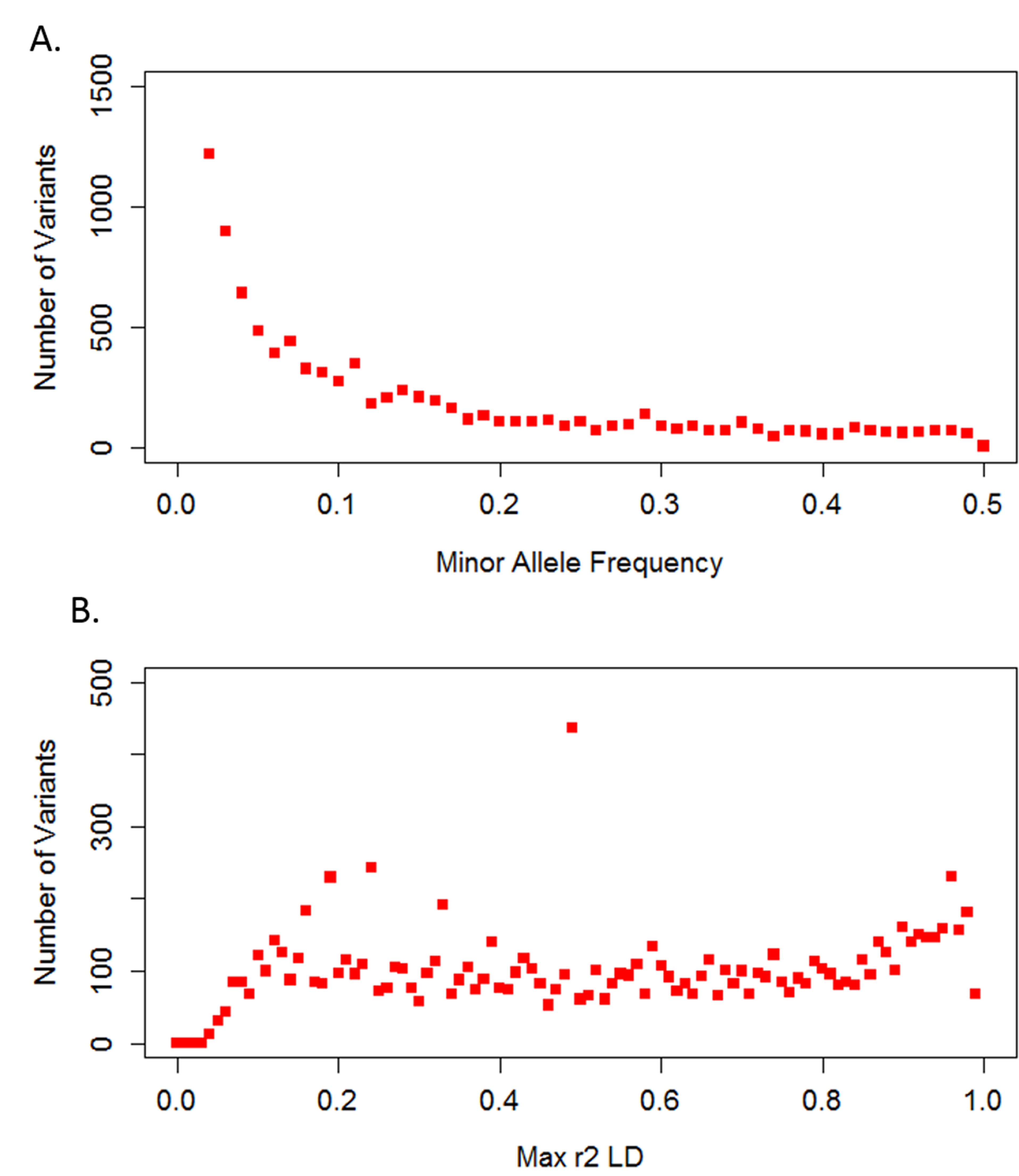

Supplement: S1 Fig — These results are for the AFR population on chromosome 15 (13,442 imputed SNPs). (TIF) [file pone.0137601.s001.tif]

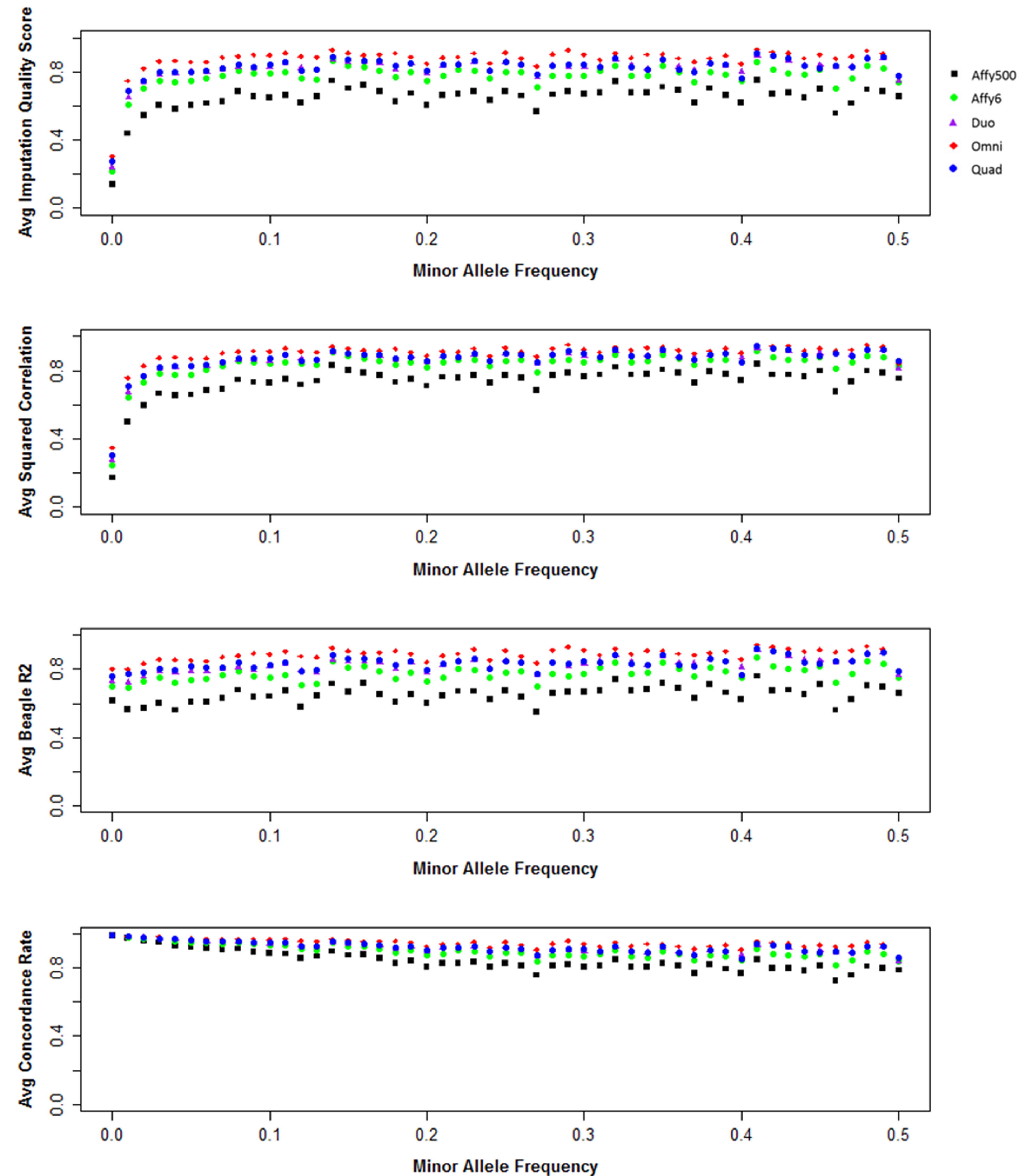

Supplement: S2 Fig — These results were produced by using the 1000 Genomes AFR reference populations as the study samples for chromosome 15. (TIF) [file pone.0137601.s002.tif]

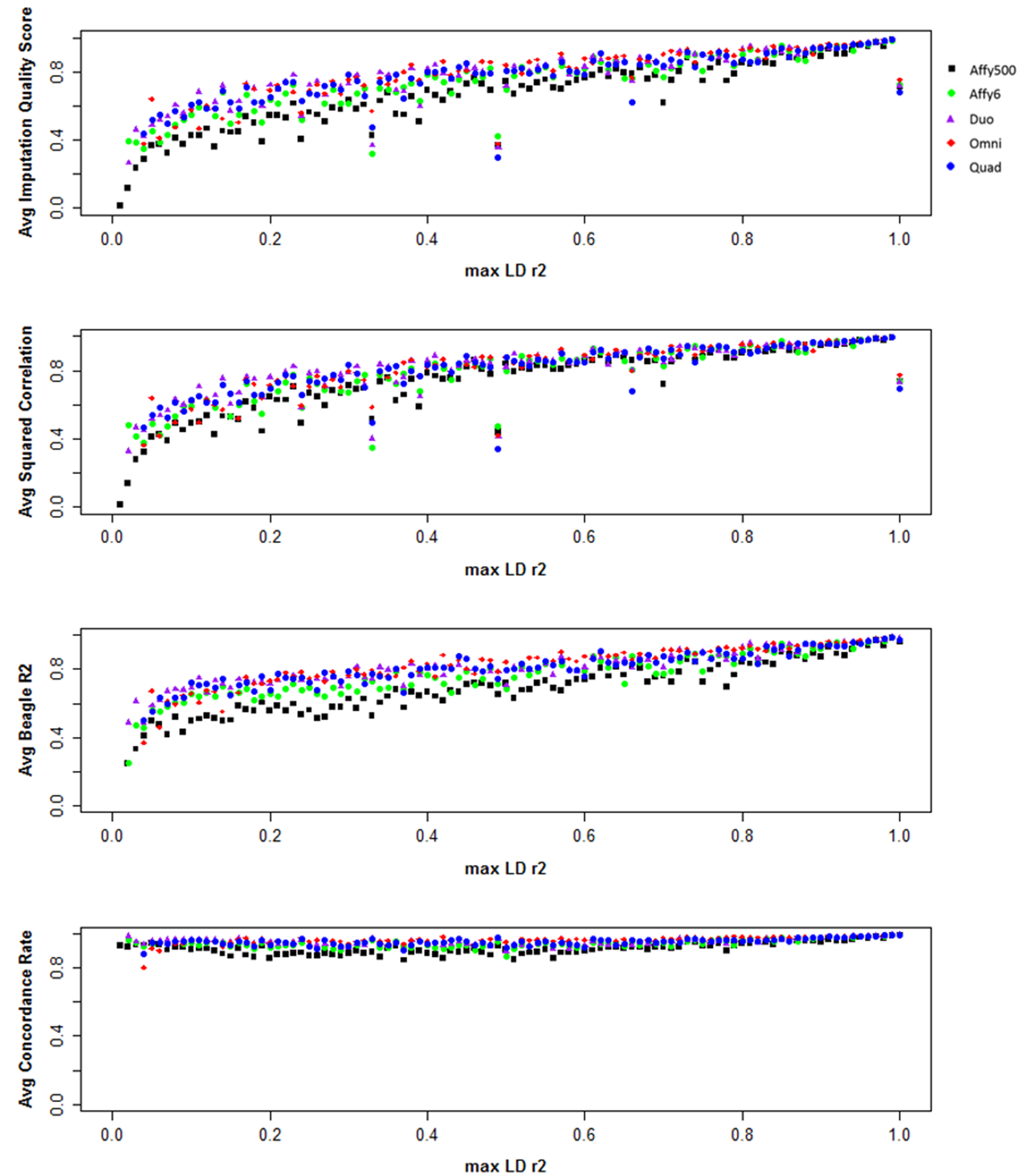

Supplement: S3 Fig — These results were produced by using the 1000 Genomes AFR reference population as the study sample for chromosome 15. (TIF) [file pone.0137601.s003.tif]

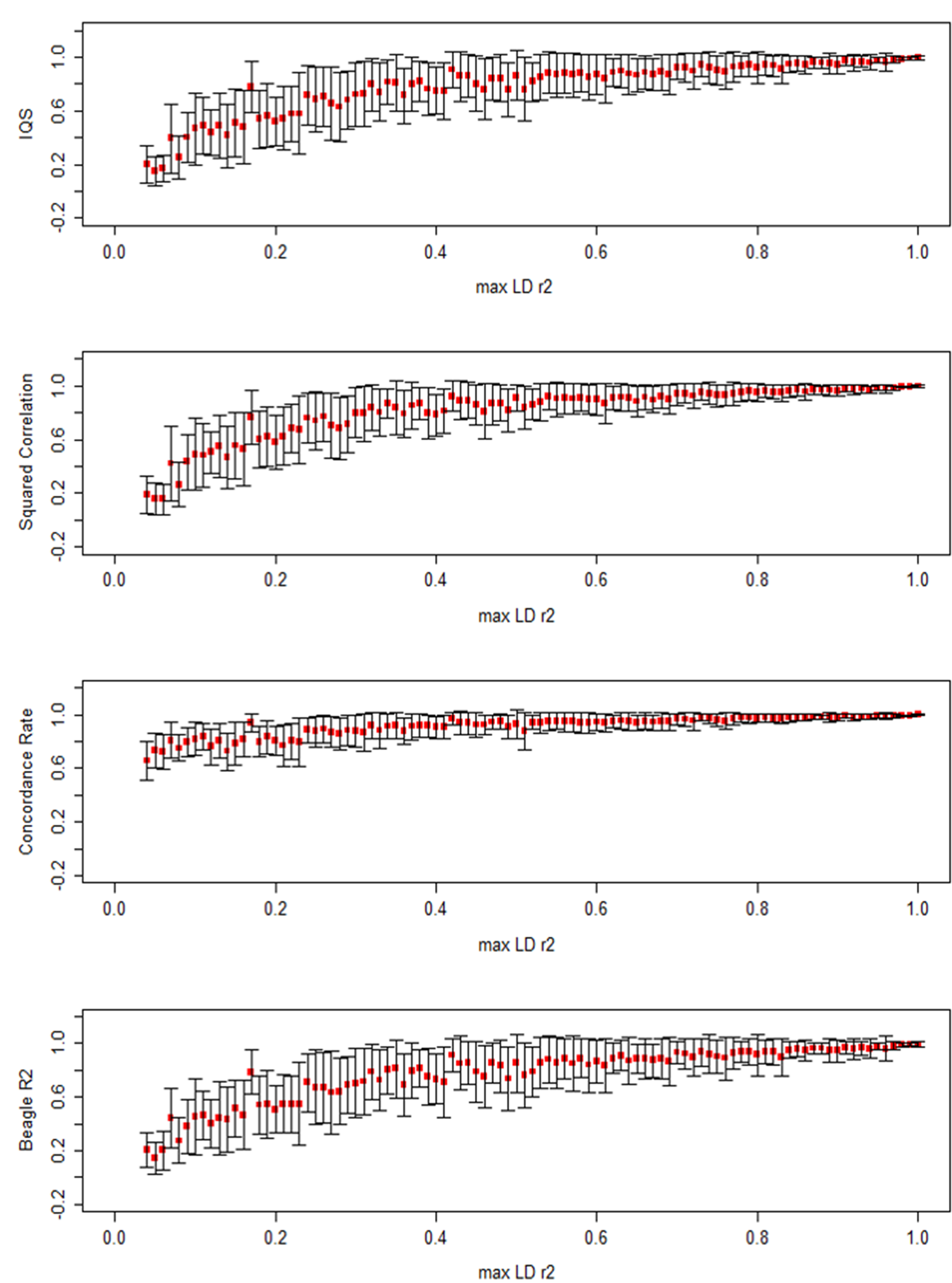

Supplement: S4 Fig — Bins are defined by 0.01 increments. Mean accuracy is denoted by the red dots and the bars indicate one standard deviation (above and below the mean). These results were produced by using 1000 Genomes AFR reference population as the study sample with Omni 2.5M typed coverage on chromosome 15. (TIF) [file pone.0137601.s004.tif]

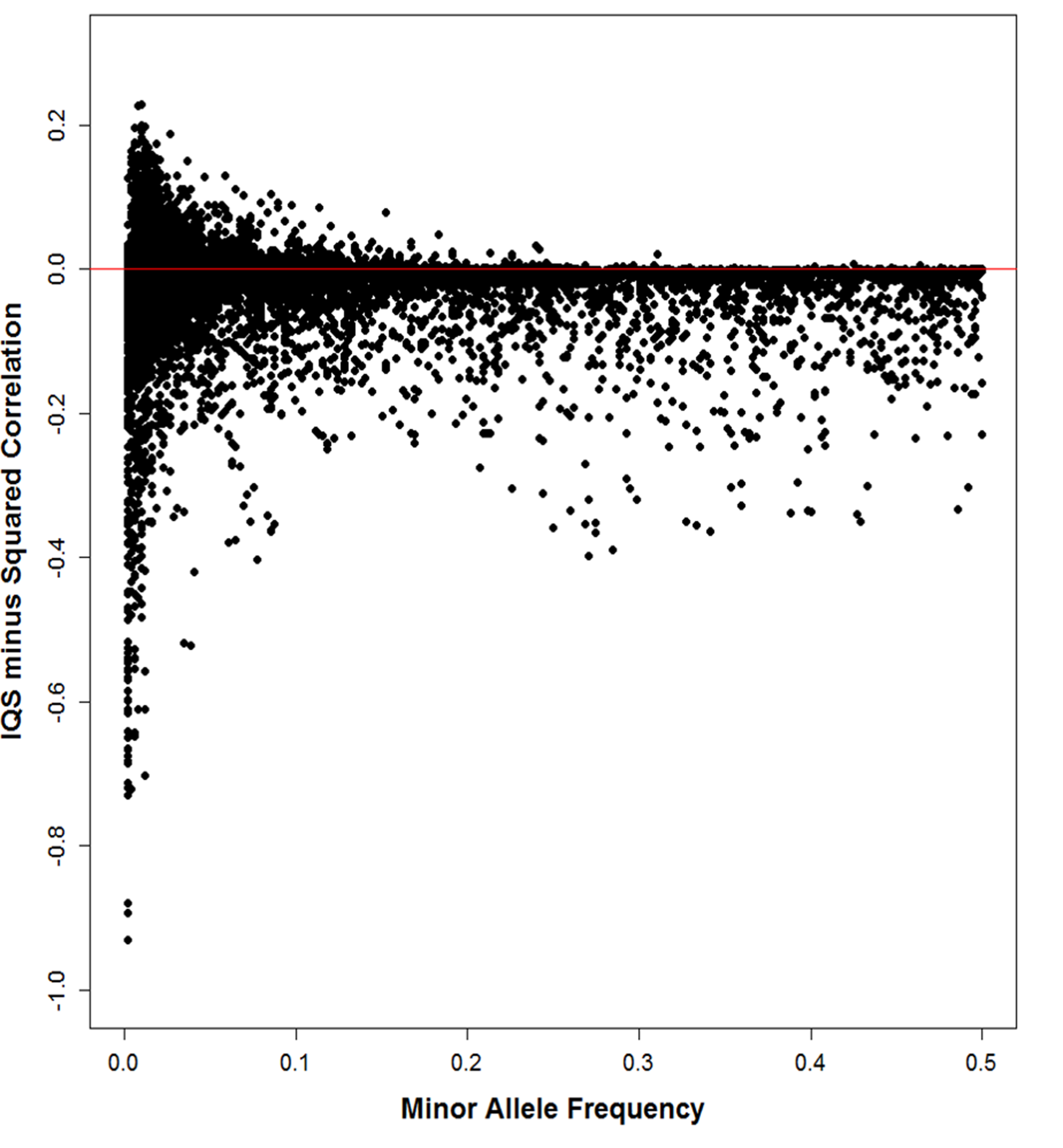

Supplement: S5 Fig — Squared correlation was subtracted from IQS for variants on chromosome 15 in the 1000 Genomes AFR reference population (N = 13,442 variants) as the study sample. Negative values indicate that the squared correlation score was higher while the positive values indicate that the IQS value was higher. The red line indicates the line y = 0. (TIF) [file pone.0137601.s005.tif]

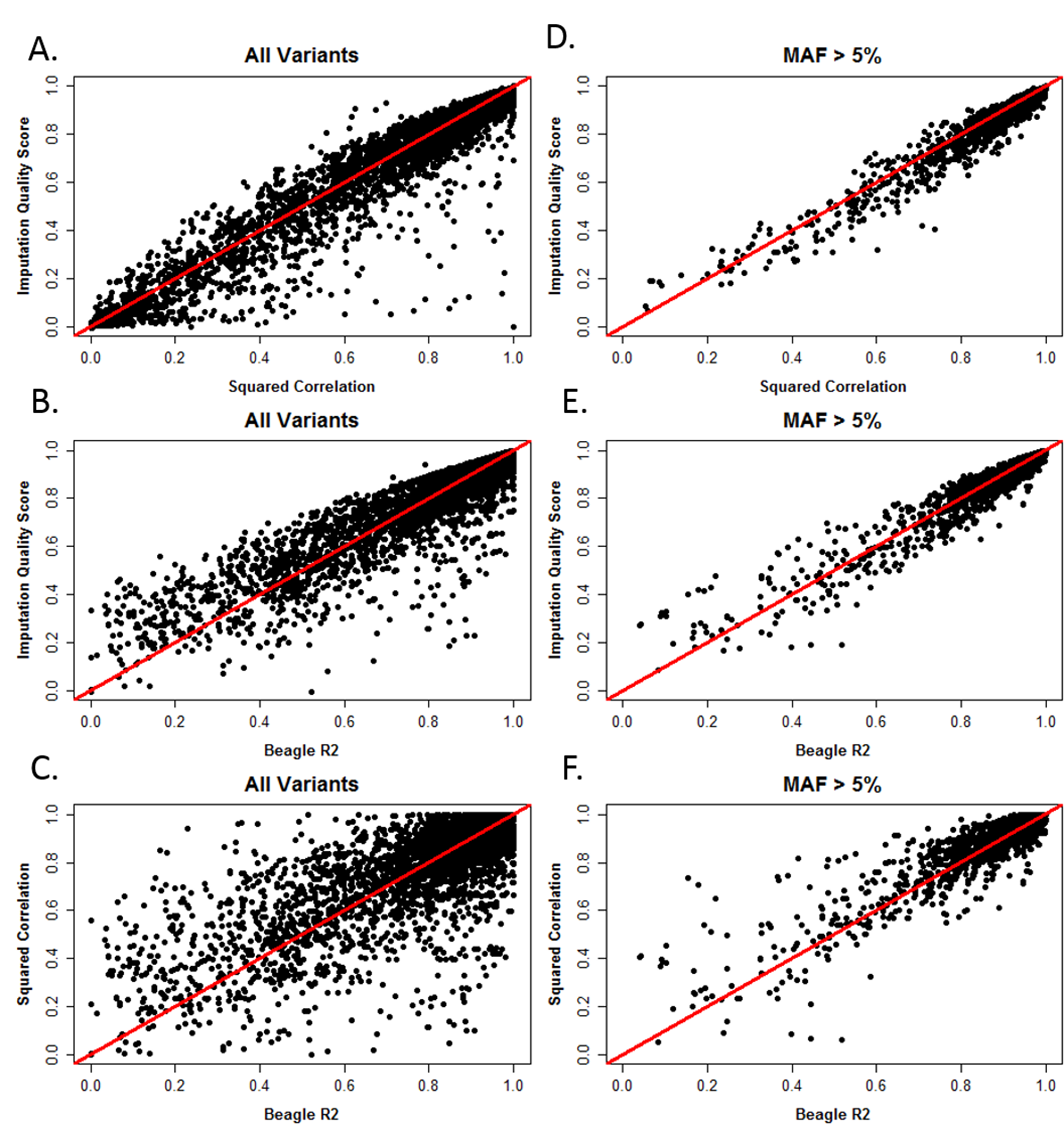

Supplement: S6 Fig — Data for all 10,937 variants are displayed in panel A, B, and C while the results for variants with MAF>5% (N = 4,533) are found in panel D, E, and F. These results were generated using Omni SNP coverage. The line y = x is denoted in red. (TIF) [file pone.0137601.s006.tif]

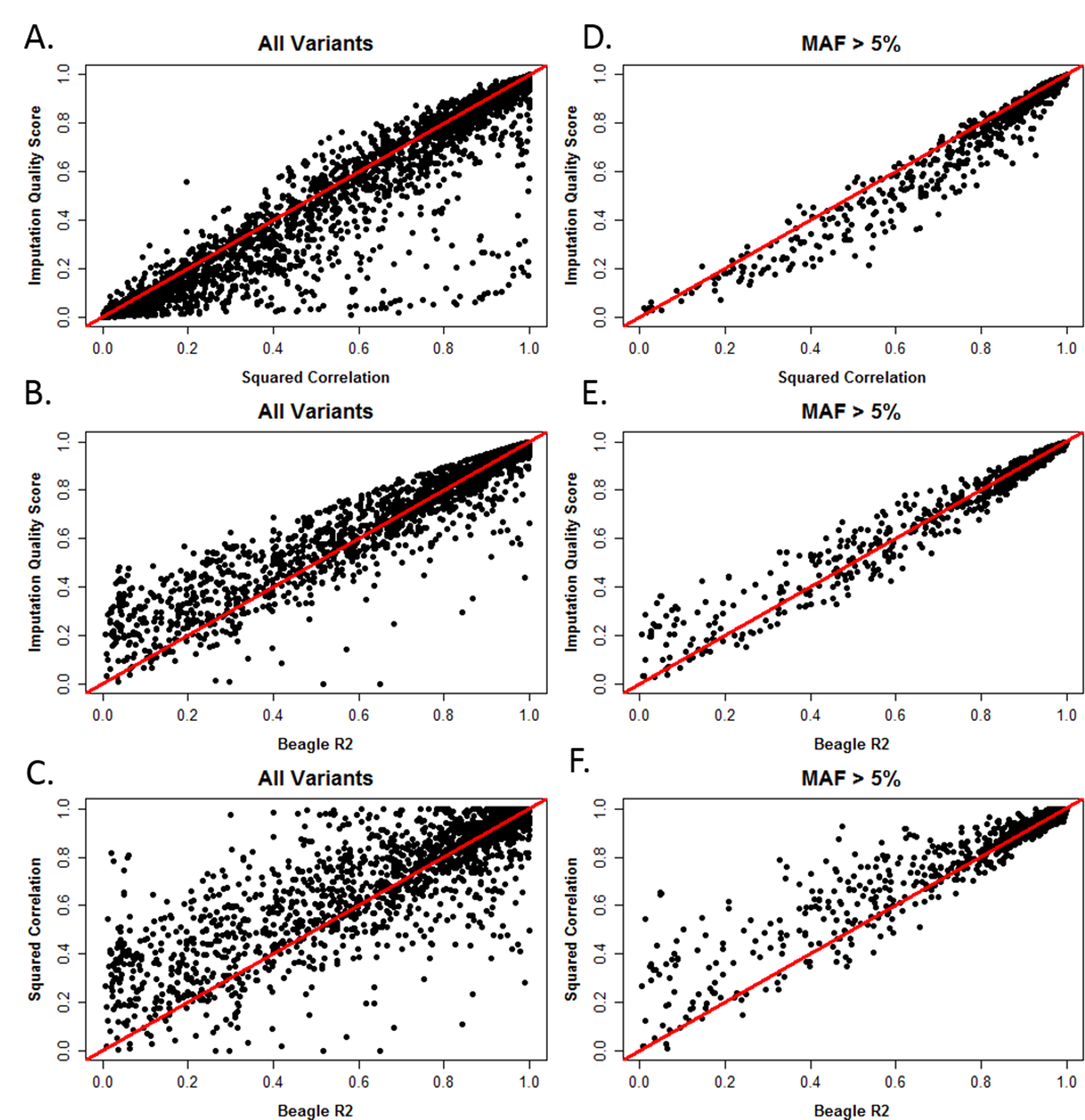

Supplement: S7 Fig — Data for all 9,401 variants are displayed in panel A, B, and C while the results for variants with MAF>5% (N = 4,627) are found in panel D, E, and F. These results were produced by using Omni SNP coverage. The line y = x is denoted in red. (TIF) [file pone.0137601.s007.tif]

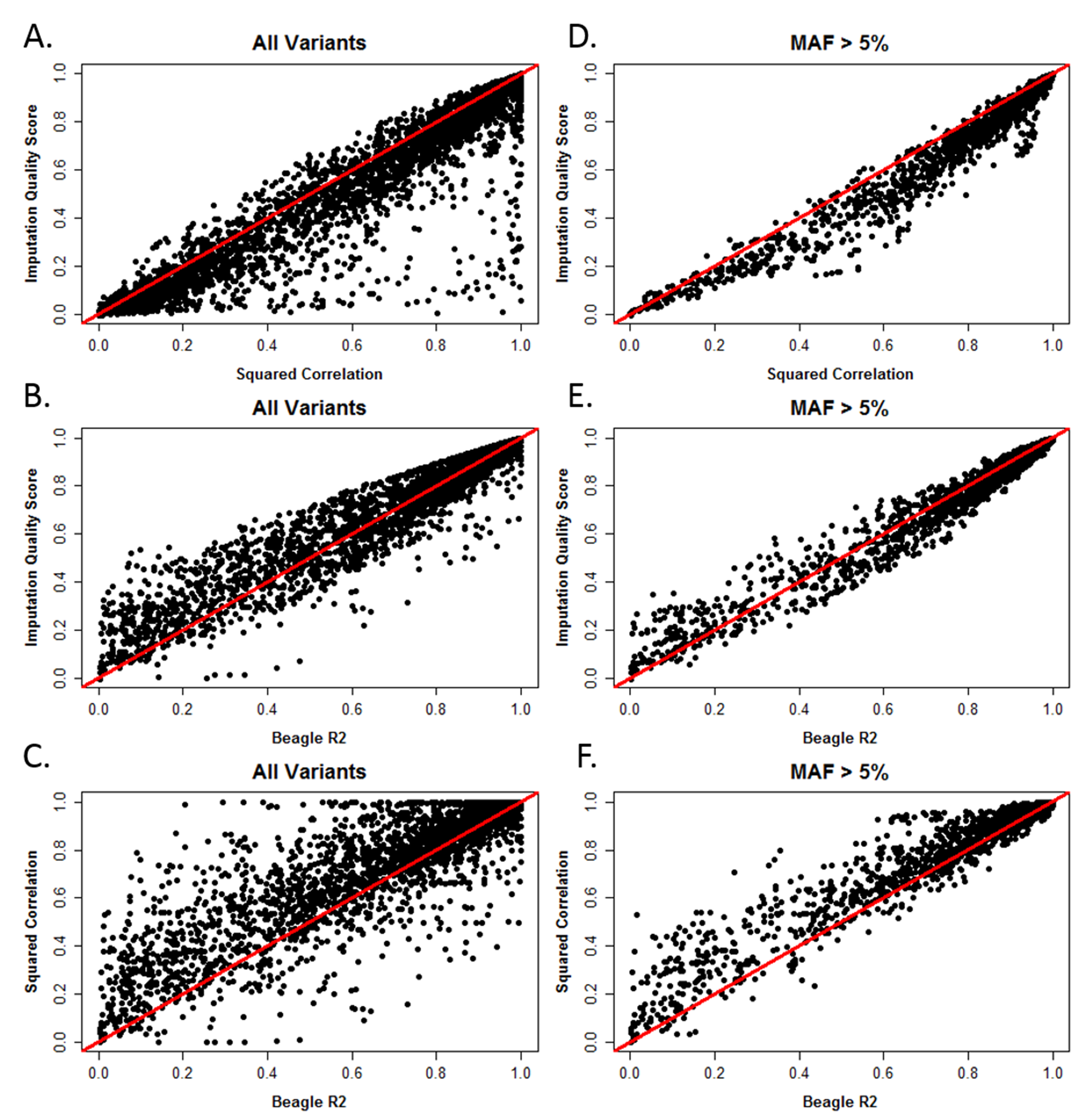

Supplement: S8 Fig — Data for all 7,401 variants are displayed in panel A, B, and C while the results for variants with MAF>5% (N = 1,903) are found in panel D, E, and F. These results were produced by using Omni SNP coverage. The line y = x is denoted in red. (TIF) [file pone.0137601.s008.tif]

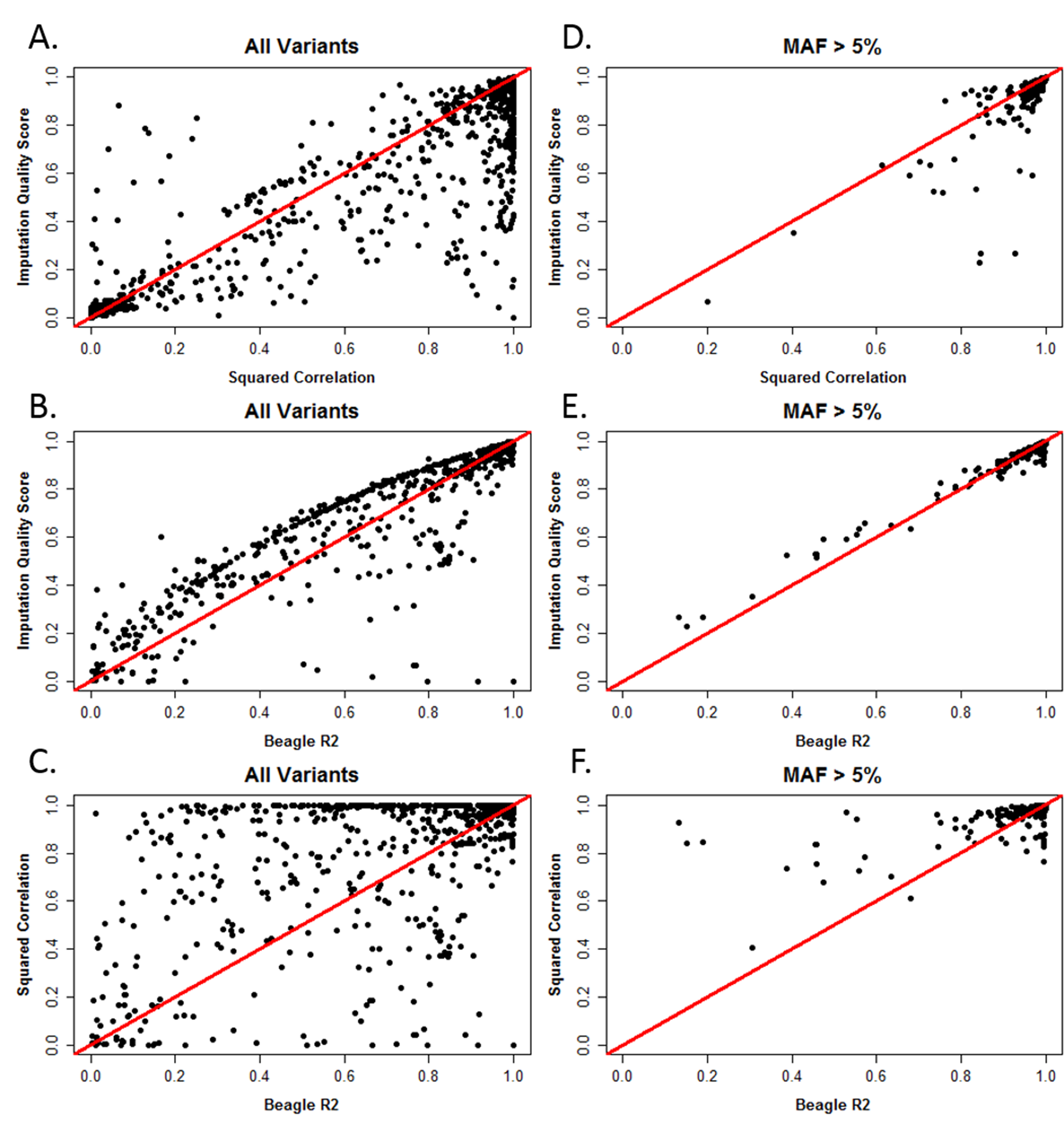

Supplement: S9 Fig — Data for all 1,170 variants are displayed in panel A, B, and C while the results for variants with MAF>5% (N = 387) are found in panel D, E, and F. These results were produced by using Omni SNP coverage. The line y = x is denoted in red. (TIF) [file pone.0137601.s009.tif]

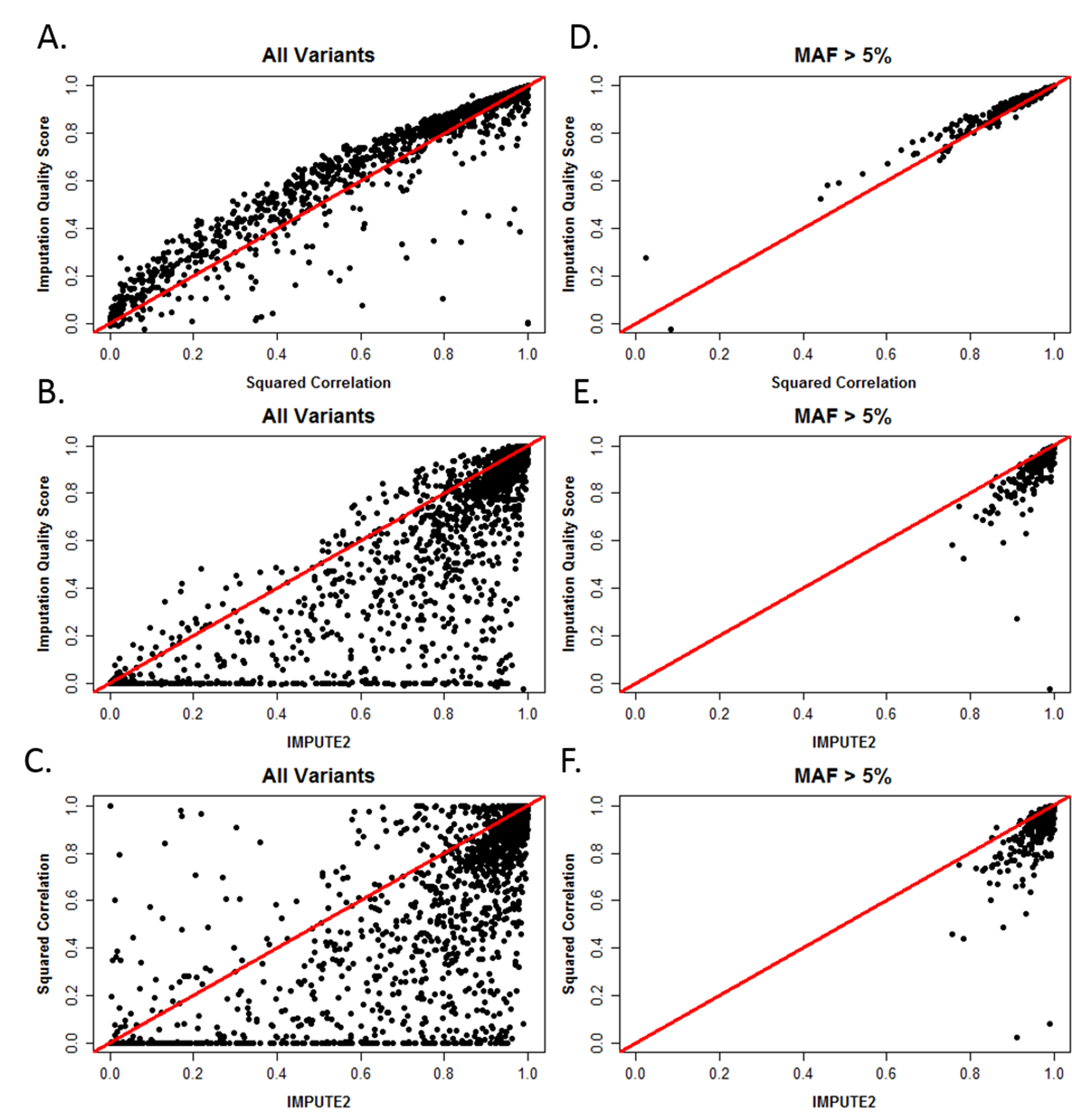

Supplement: S10 Fig — Data for all 1,878 variants are displayed in panel A, B, and C while the results for variants with MAF>5% (N = 475) are found in panel D, E, and F. These results were generated using Omni SNP coverage. The line y = x is denoted in red. (TIF) [file pone.0137601.s010.tif]

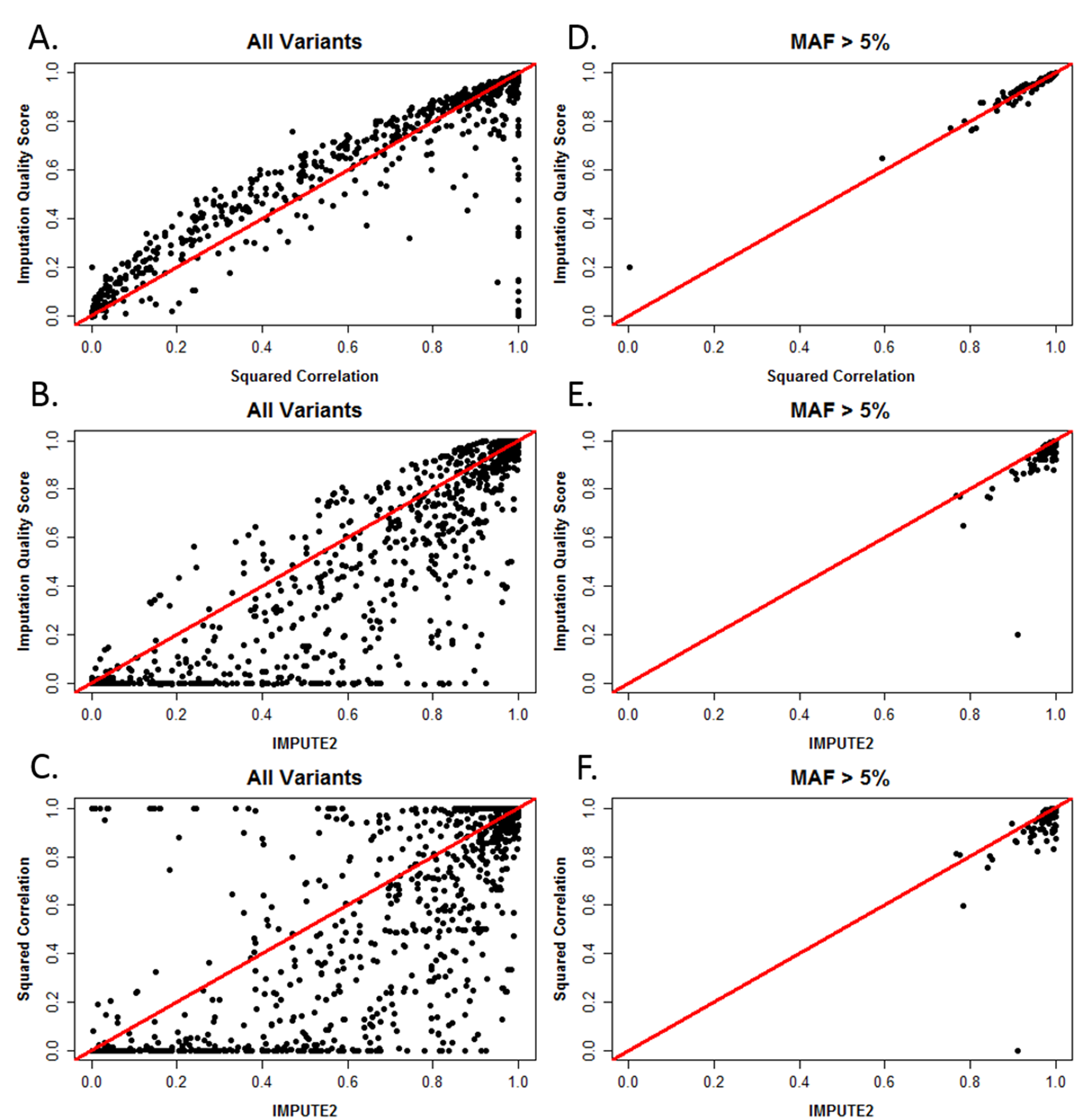

Supplement: S11 Fig — Data for all 1,253 variants are displayed in panel A, B, and C while the results for variants with MAF>5% (N = 259) are found in panel D, E, and F. These results were generated using Omni SNP coverage. The line y = x is denoted in red. (TIF) [file pone.0137601.s011.tif]
