# Supplementary material for: When Does Choice of Accuracy Measure Alter Imputation Accuracy Assessments?
Source: PLoS One. 2015 Oct 12;10(10):e0137601. doi: 10.1371/journal.pone.0137601 (PMC4601794; doi:10.1371/journal.pone.0137601)
Supplement: S1 Table — (PDF) [file pone.0137601.s012.pdf]

S1 Table. Sub-populations in the BEAGLE AFR and EUR reference panels.

AFR: 246 individuals	EUR: 379 individuals
61 African Ancestry in Southwest US (ASW)	85 Utah residents (CEPH) with Northern and Western European ancestry (CEU)
97 Luhya in Webuye, Kenya (LWK)	89 British from England and Scotland (GBR)
88 Yoruba in Ibadan, Nigeria (YRI)	98 Toscani in Italia (TSI)
	93 Finnish from Finland (FIN)
	14 Iberian populations in Spain (IBS)
