# Supplementary material for: When Does Choice of Accuracy Measure Alter Imputation Accuracy Assessments?
Source: PLoS One. 2015 Oct 12;10(10):e0137601. doi: 10.1371/journal.pone.0137601 (PMC4601794; doi:10.1371/journal.pone.0137601)
Supplement: S2 Table — Study sample variants were those found on each commercially available SNP array for the 2 MB chromosomal regions of interest. Only variants with dbSNP identifiers are listed in the number of variants in the reference panel column. (PDF) [file pone.0137601.s013.pdf]

S2 Table. Numbers of SNPs in the study samples. Study sample variants were those found on each commercially available SNP array for the 2 MB chromosomal regions of interest. Only variants with dbSNP identifiers are listed in the number of variants in the reference panel column.

Number of Genotyped SNPs in Each Region					
Chromosome	Omni 2.5M	Affy 500	Affy 6	Duo	Quad
8	1669	255	531	960	611
15	2740	555	1105	1231	1970
