# Supplementary material for: When Does Choice of Accuracy Measure Alter Imputation Accuracy Assessments?
Source: PLoS One. 2015 Oct 12;10(10):e0137601. doi: 10.1371/journal.pone.0137601 (PMC4601794; doi:10.1371/journal.pone.0137601)
Supplement: S3 Table — These variants were found in the 2 MB chromosomal regions of interest using 1000 Genomes as the study sample and were imputed using Omni 2.5 coverage. (PDF) [file pone.0137601.s014.pdf]

S3 Table. Polymorphic, imputed SNPs used in the comparison of accuracy measures. These variants were found in the 2 MB chromosomal regions of interest and were imputed using Omni 2.5 coverage.

	AFR	EUR
Chr 8	10,149	6,753
Chr 15	12,290	8,464
